# Neutrophil lymphocyte ratio as an indicator for disease progression in Idiopathic Pulmonary Fibrosis

**DOI:** 10.1136/bmjresp-2022-001202

**Published:** 2022-06-16

**Authors:** Andrew Achaiah, Amila Rathnapala, Andrea Pereira, Harriet Bothwell, Kritica Dwivedi, Rosie Barker, Valentina Iotchkova, Rachel Benamore, Rachel K Hoyles, Ling-Pei Ho

**Affiliations:** 1 MRC Immunology Unit, Weatherall Institute of Molecular Medicine, Oxford, UK; 2 Centre for Respiratory Medicine, Oxford University Hospitals NHS Foundation Trust, Oxford, UK; 3 Undergraduate Education, Great Western Hospitals NHS Foundation Trust, Swindon, UK; 4 Department of Radiology, Oxford University Hospitals NHS Foundation Trust, Oxford, UK; 5 Oxford Centre for Respiratory Medicine, Churchill Hospital, Oxford, UK

**Keywords:** interstitial fibrosis, neutrophil biology, innate immunity

## Abstract

**Rationale:**

Idiopathic pulmonary fibrosis (IPF) is a progressive fibrotic lung disease. Patients present at different stages and disease course is varied. Blood monocytes have been linked to all-cause mortality, and neutrophils to progression to IPF in patients with the indeterminate for usual interstitial pneumonia CT pattern.

**Objective:**

To determine association between blood monocytes, neutrophils and lymphocytes levels (and their derived indexes), with lung function decline and mortality in IPF.

**Methods:**

We performed a retrospective analysis of an IPF cohort (n=128) who had their first clinical visit at the Oxford Interstitial Lung Disease Service between 2013 and 2017. Association between blood monocytes, neutrophils, lymphocytes and derived indexes (within 4 months of visit) and decline in forced vital capacity (FVC) and all-cause mortality were assessed using Cox proportional hazard regression analysis. Kaplan-Meier analysis was used to assess time-to-event for 10% FVC decline and mortality for patients dichotomised to high and low leucocyte counts.

**Results:**

Median length of follow-up was 31.0 months (IQR 16.2–42.4); 41.4% demonstrated FVC decline >10% per year and 43.8% died. In multivariate models (incorporating age, gender and initial FVC%), raised neutrophils, lymphopaenia and neutrophil:lymphocyte ratio were associated with FVC decline (p≤0.01); while both monocytes and neutrophil levels (and their derived indexes) were associated with all-cause mortality (p≤0.01). Kaplan-Meier analysis also showed association between neutrophils and its derived indexes but not monocyte, with FVC decline.

**Conclusion:**

Blood neutrophil and lymphopaenia are more sensitive than monocytes as prognostic indicators of disease progression in those with established IPF.

**WHAT IS ALREADY KNOWN ON THIS TOPIC**

- Idiopathic pulmonary fibrosis (IPF) is a progressive fibrotic condition. Recently, several human studies have identified association between blood monocyte level and mortality. However, association between other leucocytes components of the ‘Full blood count’ with adverse outcomes in IPF has not been fully explored.

**WHAT THIS STUDY ADDS**

- In this study, we compared the association between blood monocytes, neutrophils and lymphocytes, measured near the presentation of disease against the specific outcome of forced vital capacity. Levels of blood neutrophil and lymphocytes but not monocytes were associated with more rapid lung function decline. Blood monocyte levels were associated with all-cause mortality.

**HOW THIS STUDY MIGHT AFFECT RESEARCH, PRACTICE AND/OR POLICY**

- These simple and easy to measure components of the full blood count have potential utility as biomarkers for lung function decline in IPF. This should be taken forward by prospective and validation studies.

## Background

Idiopathic pulmonary fibrosis (IPF) is a distinctive and progressive fibrotic condition characterised by usual interstitial pneumonia (UIP) pattern on thoracic CT. Despite advances in treatment, prognosis remains poor with a median survival of 2–4 years from diagnosis.^1^

The clinical course of IPF remains difficult to predict. Affected individuals display differing patterns of progression.^2^ This has created an urgent need for reliable, readily accessible and cost-effective biomarkers to identify individuals at greater risk of progressive disease. Most of the research efforts to date on prognostication in IPF have focused on physiological parameters,^3–6^ but there is a need to increase the repertoire of biomarkers to provide a more personalised management strategy for individual patients, better selection of patients for clinical trials and potentially widen clinical trial end points.^7^

Recently, several human studies in immunological drivers of IPF and blood-based leucocyte levels have identified monocytes and neutrophils as potential candidates for biomarkers of progressive disease. Fraser *et al* showed that monocyte levels were associated with the amount of fibrosis on CT scan,^8^ and large cohort studies from Scott linked blood monocyte levels to all-cause mortality,^4^ while Kreuter found an association with IPF progression (defined as ≥10% absolute decline in forced vital capacity (FVC%) predicted, ≥50 m decline in 6-min-walk distanc or death) within a year.^9^ In a smaller study, we found that neutrophil levels were highly significantly associated with progression of indeterminate UIP to diagnosis of IPF.^10^ These findings are supported by increasing understanding of the immune pathogenesis of IPF where innate immune cells like monocytes, monocyte-derived macrophages and neutrophils are likely to have a role in promoting fibroblast activity.^11^

In this study, we compared the association between blood monocytes, neutrophils and lymphocytes, measured near the presentation of disease against the specific outcome of FVC over a longer period of time (median 31 months) and mortality in patients with IPF. We also investigated derived indexes like neutrophil to lymphocyte ratio (NLR), monocyte to lymphocyte ratio (MLR) and Systemic Inflammation Response Index (SIRI; monocyte × neutrophils/lymphocytes) as they have been shown to be more predictive of outcome in some chronic inflammatory conditions in comparison to individual cell levels.^12^ Using multivariate Cox proportional hazard regression analysis, we demonstrated that in a model which incorporated age, gender, starting lung function and different individual leucocyte levels, blood neutrophil and lymphocyte but not monocytes levels were associated with lung function decline, while both neutrophils and monocytes were associated with all-cause mortality. We discuss the utility of blood leucocytes measured at the time of initial assessment as a prognostic indicator in IPF, and differences between our findings and recently published studies on monocyte levels and outcome in IPF.

## Methods

### Study design

**Figure 1:**
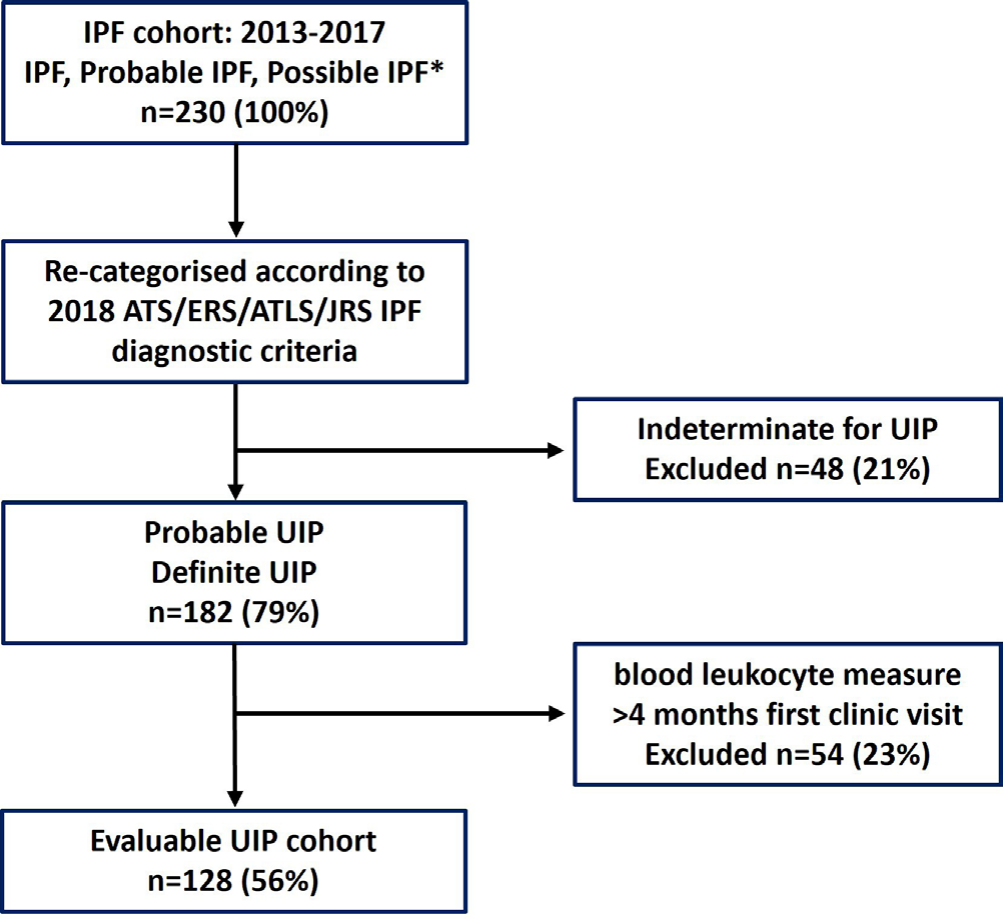
Flow diagram of radiographic progression of UIP within the IPF cohort (n=230). *Clinical diagnosis as per 2011 IPF guideline^13^ and as per 2018 IPF guideline.^14^ ATLS, Latin American Thoracic Society; ATS, American Thoracic Society; ERS, European Respiratory Society; IPF, Idiopathic pulmonary fibrosis; JRS, Japanese Respiratory Society; UIP, usual interstitial pneumonia.

We performed a retrospective analysis of a cohort of patients with IPF, probable IPF and possible IPF (defined according to the 2011, or 2018 IPF diagnostic guidelines),^13 14^ who presented to the Oxford Interstitial Lung Disease (ILD) Service between 2013 and 2017. Radiologist reports from all available thoracic CTs (including first CTs before 2013) up to August 2019 were reanalysed and cross-checked against reports from ILD multidisciplinary team meetings (MDT). Those characterised according to the 2011 guidelines were recategorised according to the 2018 IPF guideline and CT scans previously labelled as ‘possible UIP’, were recategorised as ‘probable UIP’ or ‘indeterminate for UIP’.^14^ Patients with a baseline CT scan compatible with ‘probable UIP’ and ‘definite UIP’ were included in the study, while those with indeterminate for UIP pattern were excluded (figure 1).

The following data were collected—patient demographics, date of first and follow-on CTs, date of diagnosis of IPF (MDT), all available pulmonary function tests and additional comorbidities. Neutrophils, lymphocytes and monocyte levels performed using standard ‘full blood count’ analysis measured within 4 months of presentation to our clinics were included. NLR, MLR and SIRI, the product of neutrophil and monocyte values divided by lymphocyte values,^15^ were also calculated.

To characterise our cohort, we compared the initial thoracic CT with the latest in the 5-year follow-up period, in a subset of patient who had follow-on CTs after the initial CT (n=82).

A measured reduction in absolute FVC (litres)>10% per year was considered clinically significant and representative of physiological decline.

### CT scan

CT scans were acquired using a 64-detector row CT scanner (LightSpeed VCT XT; GE Medical Systems, Milwaukee, Wisconsin, USA). Images were reconstructed using a high spatial resolution algorithm. A volumetric scan was performed with 0.625 mm slice thickness at an interval of 0.625 mm. All CT abnormalities were defined using standard Fleischner-based terminology.^16^ Definitions of CT patterns were according to 2018 IPF guidelines. Briefly, ‘definite UIP’ pattern was defined by the presence of a basal-predominant subpleural reticular abnormality and honeycombing with or without traction bronchiectasis. Probable UIP pattern was defined as basal and subpleural predominant reticulation and traction bronchiectasis or bronchiolectasis with or without mild ground glass opacification (GGO). ‘Indeterminate for UIP’ was defined as subtle reticulation in the presence or absence of mild GGO and with a basal/subpleural predominant distribution.

### Patient and public involvement

Patient and public were not involved in design, recruitment, conduct of this study.

### Statistical analysis

Data are expressed as absolute values, relative percentages, means (with SD), medians (IQR) or by dichotomised value. Normality tests were performed using a D'Agostino & Pearson test. Difference between groups was analysed using Student’s t-tests or Mann-Whitney U test for parametric and non-parametric analysis, respectively. Contingency tests (Fisher’s exact test of significance) were used to assess categorical data. Kaplan-Meier analysis (log rank test of significance) was performed to evaluate time-to-event from first clinic assessment for hospitalisation and survival.

We employed a multivariate Cox proportional hazard regression analysis incorporating age, gender, starting lung function and different individual leucocyte levels in the model to test association between blood monocyte, neutrophil and lymphocyte and the clinical outcomes of FVC decline >10% per year or mortality. Neutrophil, monocyte, and lymphocyte counts were analysed in continuous, or dichotomised values either as above or below upper limit of normal range (monocyte <or >0.90 x10^9^/L, neutrophil; <or >7.5 x10^9^/L) or lower (lymphocyte; <or > 1.0 x10^9^/L) or above or below median values for NLR, MLR and SIRI. Time-dependent effects were included in the model, and proportional hazards assumption was tested for each. Hazard ratios generated for continuous covariates represent the change in the risk of outcome if the covariate in question changes by one unit. HRs generated for dichotomised covariates represents the risk of achieving outcome if the covariate is present. Statistical significance was performed using the likelihood ratio test as reported by the coxph function at the 95% significance level. All analyses were performed using Graphpad Prism (V.9) apart from Cox proportional hazard modelling which was performed with R Studio (V.3.6.2) statistical programming language packages Survival (V.3.1.8) and Survminer (V.0.4.6). Reported p values were two sided and a p<0.05 was considered significant.

## Results

### Cohort characteristics and clinical outcomes

A total of 230 individual patients with a clinical diagnosis of IPF (according to 2011 criteria) were identified. In 48 cases (20.9%), thoracic CT at time of initial ILD assessment was consistent with ‘Indeterminate for UIP’ pattern and excluded. Of the remaining 182 patients, 54 (29.6%) patients did not have an available blood leucocyte measurement within 4 months of initial ILD assessment and were excluded (figure 1). The final cohort consisted of 128 patients; 55% had ‘probable UIP’ on CT scanning and 45% had definite UIP.

Baseline demographic data, physiological indices, blood leucocyte levels, comorbidity profiles and imaging features for the final cohort are shown in table 1. Mean age was 75.2±7.8 years, 78% were male. Median length of follow-up was 31.0 months (16.2–42.4). Median time between blood leucocyte collection and first ILD clinic assessment was 0 days (IQR 0–17 days); all blood samples were taken before or after initial visit to clinic, and all within 4 months. Seventy patients (55%) had blood taken within 7 days of assessment.

**Table 1:** Characteristics for patients, at the point of initial CT when IPF was first diagnosed; all patients divided on presence of radiographic progression on follow on CT

	All patients (n=128)	Patients categorised by FVC decline		P value	OR
		FVC decline <10%/ year (n=62)	FVC decline ≥10%/ year (n=53)		
Demographics					
Female (%)	28 (21.9)	13 (21.0)	14 (26.4)	0.516	1.35
Male (%)	100 (78.1)	49 (79.0)	39 (73.6)		0.74
Age at first clinic visit (SD)	75.22 (7.88)	74.8 (6.9)	74.4 (8.6)		
Smoking status					
Ex-smoker	77 (60.2)	43 (69.4)	26 (49.1)	0.113	0.45
Never	30 (23.4)	12 (19.4)	16 (30.2)		
No data	21 (16.4)	7 (11.2)	11 (20.7)	--	--
GAP index					
1	66 (57.4)	39 (67.2)	26 (54.2)	0.229	0.58
2	39 (33.9)	15 (25.9)	19 (39.6)	0.148	1.87
3	10 (8.7)	4 (6.9)	3 (6.3)	>0.999	0.90
Comorbidity					
Type II diabetes mellitus	23 (18)	12 (19.4)	8 (15.1)	0.626	0.74
Gastro-oesophageal reflux	16 (12.5)	7 (11.3)	8 (15.1)	0.588	1.40
Ischaemic heart disease	20 (15.6)	9 (14.5)	10 (18.9)	0.618	1.37
Atrial fibrillation	9 (7)	2 (3.2)	6 (11.3)	0.141	3.83
COPD	14 (10.9)	5 (8.1)	8 (15.1)	0.255	2.03
Blood leucocytes					
Monocyte (×10^9^ /L)	0.70 (0.56–0.84)	0.67 (0.56–0.80)	0.70 (0.53–0.81)	0.925	
Lymphocyte (×10^9^ /L)	1.76 (1.41–2.40)	1.81 (1.56–2.50)	1.65 (1.40–2.32)	0.206	
Neutrophil (×10^9^ /L)	5.25 (3.96–6.68)	5.23 (3.90–6.31)	5.23 (4.30–7.06)	0.190	
Leucocyte-derived indexes					
NLR	2.77 (1.96–3.85)	2.46 (1.87–3.55)	3.17 (2.09–4.21)	0.049	
MLR	0.37 (0.31–0.50)	0.35 (0.30–0.41)	0.42 (0.30–0.53)	0.151	
SIRI	2.00 (1.22–2.83)	1.71 (1.10–2.49)	2.18 (1.32–2.99)	0.089	
Pulmonary function tests					
%TLCO	57.7 (47.5–69.0)	61.9 (50.9–71.0)	55.5 (47.3–68.4)	0.233	
%FVC	81.0 (68.1–98.3)	85.5 (69.9–98.0)	79.4 (70.1–101.2)	0.667	
CPI Score^27^	68.8 (61.3–76.1)	65.6 (60.9–75.7)	69.19 (62.1–74.5)	0.570	
CT pattern					
Probable UIP	71 (55.5)	38 (61.3)	25 (47.2)	0.138	0.56
Definite UIP	57 (44.5)	24 (38.7)	28 (52.8)	0.138	1.77
Antifibrotic use	56 (43.7%)	25 (40.3%)	31 (58.5%)	0.063	2.08
Corticosteroids use	11 (8.6%)	4 (6.6%)	7 (13.2%)	0.341	2.21
At baseline visit	1 (0.8%)	0 (0%)	1 (1.8%)	--	--
During follow-up	10 (7.8%)	4 (3.2%)	6 (11.3%)	0.510	1.85

Continuous variables expressed as median values (IQR) with exception of age (mean, SD).

COPD, Chronic obstructive pulmonary disease; CPI, Composite Physiological Index as calculated by Wells et al; FVC, forced vital capacity; GAP, Gender-Age-Physiology Index; IPF, idiopathic pulmonary fibrosis; MLR, monocyte:lymphocyte ratio; NLR, neutrophil:lymphocyte ratio; SIRI, systemic inflammation response index; TLCO, Transfer factor for carbon monoxide; UIP, usual interstitial pneumonia.

During follow-up 56 deaths (44%) were recorded; median time from first ILD assessment to death was 25.8 months (18.4–39.8) compared with 45.1 months (37.8–56.0) in those alive at time of censoring (01/08/2019). Most frequent cause of death was end-stage IPF (11%); followed by pneumonia (6.3%) and acute exacerbation of IPF (1.6%). Cause of mortality was not identified in 25 cases (19.5%).

**Figure 2:**
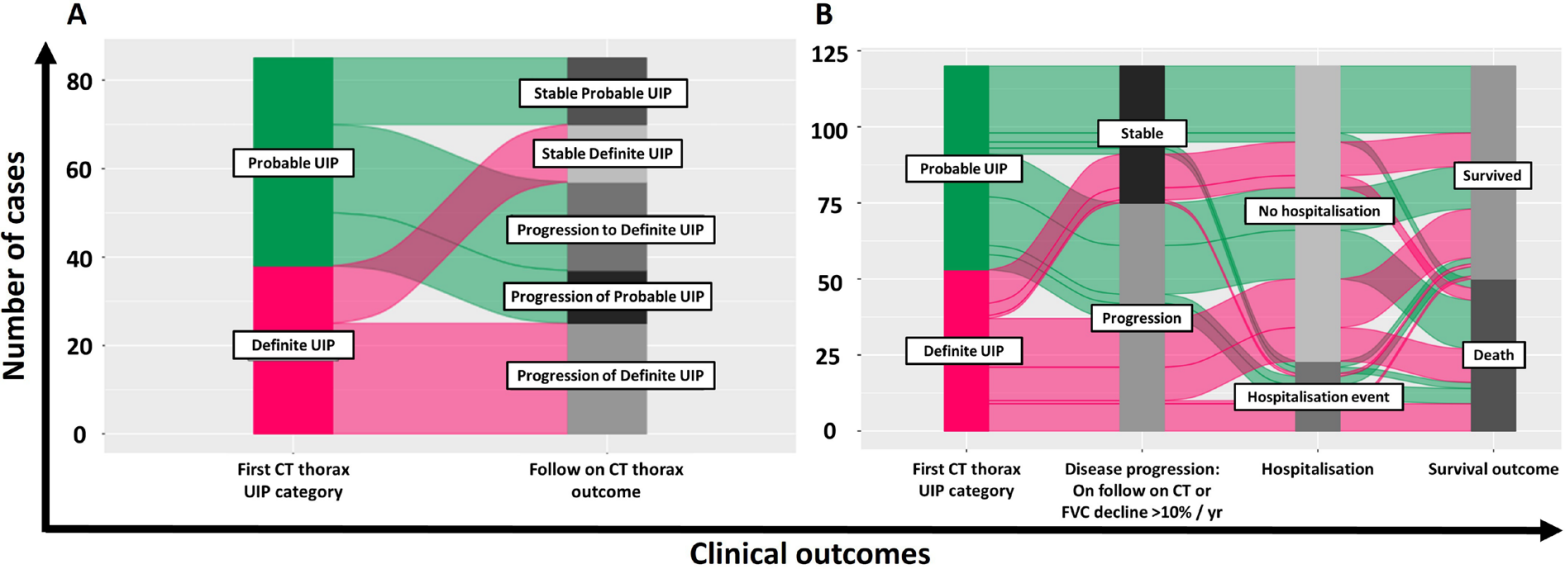
Alluvial plots demonstrating the proportion of patients per clincal outcome measure. (A) Illustrates the proportions of cases demonstrating stable and progressive radiological appearance in patients that underwent follow-on CT scan (n=86). (B) Illustrates the proportion of cases with disease progression (either on follow-on CT and/or by demonstrating FVC >10% decline/year), hospitalisation events and survival. FVC, forced vital capacity; UIP, usual interstitial pneumonia.

All 128 patients had CT scans; 86 had a follow-on CT within 5 years of the first CT scan. 55.5% of the initial CT scans demonstrated probable UIP and 45%, definite UIP. Radiographic progression was observed in 58 (67.4%) cases over a median time interval of 30.8 months (21.5–44.1)—13 patients demonstrated progression in the extent of probable UIP, 20 progressed from probable UIP to definite UIP and 25 progressed in extent of definite UIP (figure 2).

Lung function decline of FVC >10% of starting FVC, per year, was identified in 46% of the cohort. Mean annualised change across all patients with available readings was −8.25% (±17.7).

### Association of neutrophils, monocytes and lymphocytes with FVC decline

Of the leucocyte variables studied using multivariate analysis, neutrophil count >7.5×10^9^/L (3.12, 1.44–6.74, p=0.004) was most associated with FVC decline over a median of 31 months (table 2). In addition, continuous neutrophil level, lymphopenia, NLR, age and being female, but not monocytes were also associated with FVC decline of greater than 10% in a year (table 2). NLR was most statistically significantly associated with FVC decline (HR 1.3; 1.16–1.48; p=0.00002).

**Table 2:** Multivariate models testing blood leucocytes against outcome

Outcome	Multivariate analysis			
	HR	95% CI	P value	P value (PH assumption)
**FVC decline >10%/year**				
Absolute leucocytes				
Gender (male)	0.48	0.24 to 0.97	0.040*	0.470
Age	1.1	1.0 to 1.15	0.035*	0.110
FVC (%)	0.99	0.97 to 1.00	0.071	0.630
Monocytes (×10^9^/L)	0.61	0.11 to 3.74	0.500	0.249
Lymphocytes (×10^9^/L)	0.81	0.52 to 1.3	0.360	0.850
Neutrophils (×10^9^/L)	1.4	1.1 to 1.7	0.0011*	0.760
Monocytosis (>0.90×10^9^/L)	1.83	0.88 to 3.82	0.105	0.953
Lymphopenia (<1.0×10^9^/L)	3.78	1.31 to 10.97	0.014*	0.669
Neutrophilia (>7.5×10^9^/L)	3.12	1.44 to 6.74	0.004*	0.870
Leucocyte indexes	**Adjusted HR**			
MLR	1.07	0.70 to 1.64	0.763	0.757
MLR >0.37	1.91	1.07 to 3.40	0.029*	0.434
NLR	1.31	1.16 to 1.48	0.00002*	0.679
NLR >2.77	2.04	1.12 to 3.71	0.020*	0.803
SIRI	1.03	0.97 to 1.08	0.326	0.578
SIRI>2.0	1.95	1.10 to 3.47	0.023*	0.549
**Mortality**				
Absolute leucocytes				
Gender (male)	0.83	0.38 to 1.80	0.630	0.970
Age	1.1	1.00 to 1.10	0.005*	0.930
Baseline FVC (%)	0.97	0.96 to 0.99	0.001*	0.990
Monocytes (×10^9^/L)	1.4	1.10 to 1.80	0.013*	0.340
Lymphocytes (×10^9^/L)	1.0	0.70 to 1.50	0.890	0.940
Neutrophils (×10^9^/L)	1.2	1.10 to 1.40	0.0008*	0.940
Monocytosis (>0.90×10^9^/L)	1.01	0.50 to 2.01	0.990	0.291
Lymphopenia (<1.0×10^9^/l)	1.50	0.52 to 4.38	0.451	0.279
Neutrophilia (>7.5×10^9^/L)	2.31	1.18 to 4.54	0.015*	0.544
Leucocyte indexes	**Adjusted HR**			
MLR	1.32	1.09 to 1.60	0.005*	0.136
MLR >0.37	2.05	1.26 to 3.74	0.019*	0.583
NLR	1.22	1.11 to 1.34	0.00006*	0.691
NLR >2.77	1.81	1.01 to 3.23	0.046*	0.767
SIRI	1.06	1.02 to 1.09	0.001*	0.130
SIRI>2.0	1.83	1.02 to 3.26	0.041*	0.231

Absolute leucocyte counts were tested in combination (absolute monocyte, lymphocyte and neutrophils) to explore interaction and adjusted for the covariates gender, age and baseline FVC%. For multivariate models exploring contribution of the leucocyte derived indexes (MLR, NLR or SIRI); each was tested individually with the covariates gender, age and baseline FVC% but HR outcome for gender, age and baseline FVC% are not shown. All adjusted covariates in each model satisfied the proportional hazard assumption (p<0.05 are considered significant).

FVC, forced vital capacity; MLR, monocyte/lymphocyte ratio; NLR, neutrophil/lymphocyte ratio; SIRI, Systemic Inflammatory Response Index.

These data support neutrophil and lymphopaenia as a correlate for progression of fibrosis in IPF.

### Association of neutrophils, monocytes and lymphocytes with all-cause mortality

Both neutrophil (HR 1.2, 95% CI 1.10 to 1.40, p<0.001) and monocyte levels (1.4, 95% CI 1.10 to 1.80, p=0.013) were significantly associated with all-cause mortality (table 2). In addition, male gender, increasing age, FVC, MLR, NLR and SIRI were also associated with mortality within 5 years of measurement.

### Greater rate of FVC decline in patients with higher neutrophil levels

**Figure 3:**
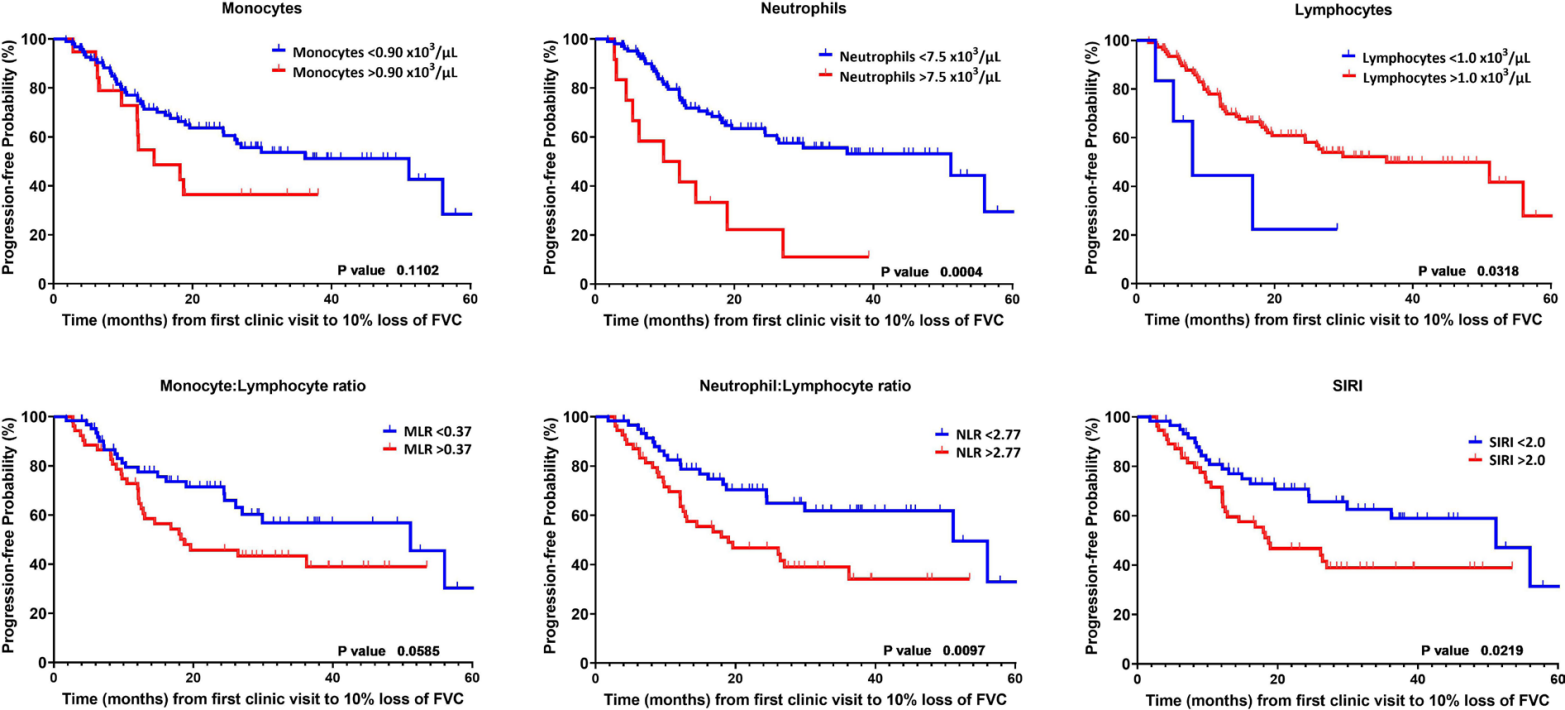
Kaplan-Meier curves for FVC decline dichotomised by normal and abnormal upper limit for leucocytes and by median values for the derived indexes of monocyte:lymphocyte ratio (MLR) (>0.37), neutrophil:lymphocyte ratio (NLR) (>2.77) and Systemic Inflammatory Response Index (SIRI) (>2.00). P value calculated with log rank test. P value calculated with log rank test. FVC, forced vital capacity.

**Figure 4:**
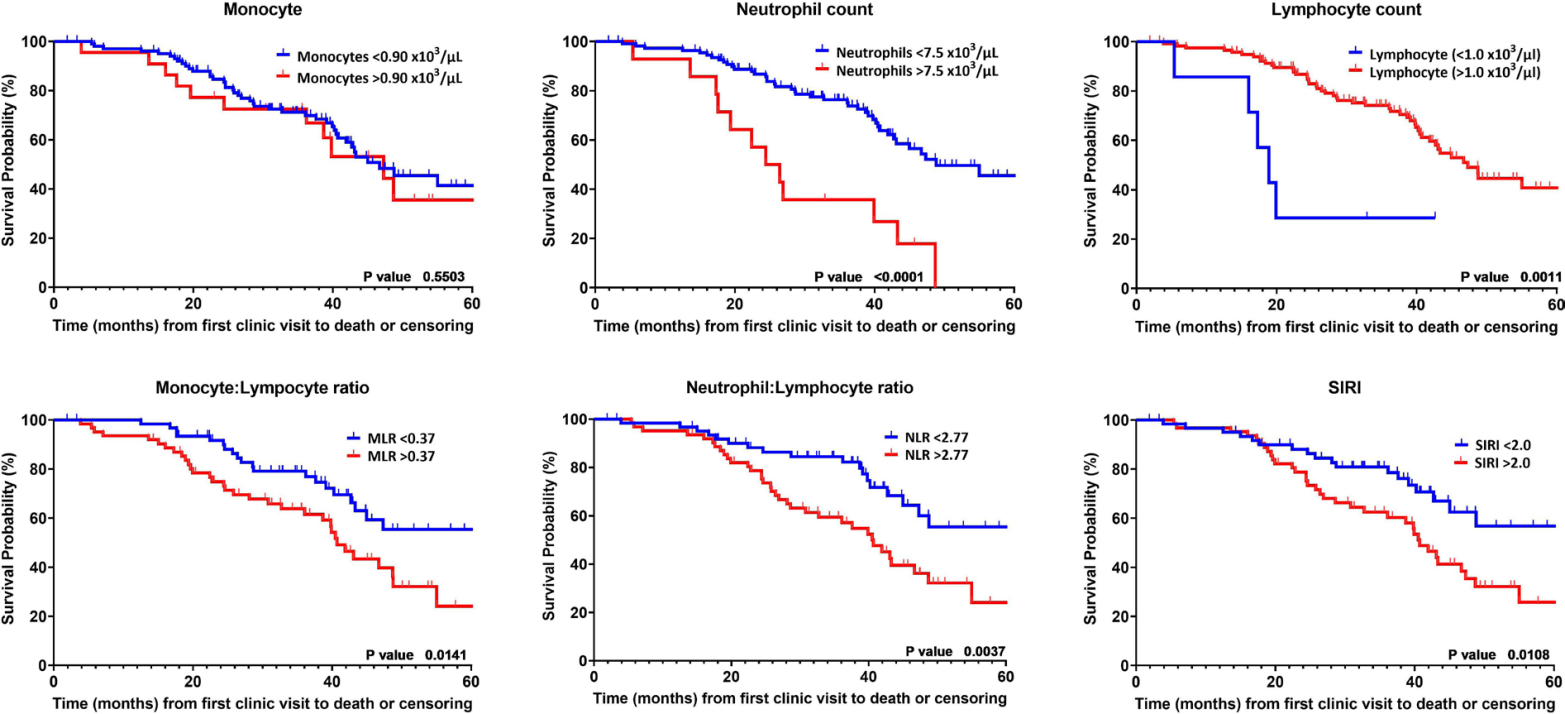
Kaplan-Meier curves for survival dichotomised by normal and abnormal upper limit for leucocytes and by median values for the derived indexes of monocyte:lymphocyte ratio (MLR) (>0.37), neutrophil:lymphocyte ratio (NLR) (>2.77) and Systemic Inflammatory Response Index (SIRI) (>2.00). P value calculated with log rank test.

Kaplan-Meier analysis also supported the findings from our multi-variate analysis. Neutrophilia, lymphopenia and NLR and SIRI; but not monocytosis or MLR were associated with more rapid lung function decline (figure 3). All leucocyte levels were dichotomised by greater or lower than the upper limit of normal. Using this dichotomy, we did not observe a significant correlate in monocytosis with mortality within 5 years (figure 4). However, neutrophilia and lymphopaenia, and the derived leucocyte indexes correlated with worse survival (figure 4).

## Discussion

In this single-centre retrospective study, a strong association was observed between neutrophil levels lymphopaenia and NLR, but not monocyte levels with FVC decline. Instead, monocytes (and neutrophils) were associated with mortality. This finding extends that from Scott and Kreuter,^4 9^ who found that monocyte levels were associated with mortality and a composite outcome measure respectively in IPF patients. There were key differences between our study and these previous publications. First, we examined all the three main parameters of the blood leucocyte profile—neutrophils, lymphocytes and monocytes while Scott and Kreuter focused on monocyte levels only. We did this in part, because of our previous findings of correlation between neutrophils and progression of iUIP to definite/probable UIP on CT scans,^10^ but also because there is ample evidence that neutrophils are involved in the immune-pathogenesis of IPF (described further below).^11^ Although Scott also examined the transcriptome of blood immune cells, these were on RNA from stored peripheral blood mononuclear cells, where the neutrophil component would not have survived storage.

The second major difference were the outcome measures. In our study, we specifically separated disease progression from all-cause mortality. We defined disease progression by relative decline in absolute FVC and/or progression in CT abnormalities to provide a more IPF-specific measure of deterioration in disease state. This complemented all-cause mortality, which in an elderly IPF cohort is likely to have other contributory factors to mortality. By contrast, in the larger studies Scott *et al*^4^ only report all-cause mortality and Kreuter *et al*^9^ define disease progression as a composite outcome of mortality, lung function decline and decline in 6 min walk test. Our study also had a longer follow-up period (median of 31 months for the cohort with a 5-year recruiting period, as opposed to 1 year for the other studies). As such we were able to calculate annualised FVC decline over a larger time period and identify FVC decline >10%/yr more accurately. Scott’s cases were also identified by International Classification of Disease-10 coding of clinical records which may pose limitation to the findings. These differences may account for the differences in findings between these studies.

Thus, while there is corroboration for the association between peripheral monocyte count and all-cause mortality observed by Scott, and with the composite outcome in Kreuter *et al*^9^ that included mortality, our study suggests that when divided into outcomes that relate more specifically to disease progression (FVC decline), neutrophil and lymphopaenia appear more sensitive compared with monocytes as a correlate.

Mechanistically, our findings could be explained by experimental evidence implicating neutrophils, monocytes and lymphocytes in immunopathogenesis of IPF. Strong evidence supports monocyte and monocyte-derived macrophages in the aetiopathogenesis lung fibrosis from animal and human studies.^17 18^ The contribution of neutrophils and lymphocytes to the pathogenesis of IPF is becoming increasingly understood. Neutrophils secrete proteases, cytokines and chemokines which can propagate inflammatory responses.^19^ Neutrophilic bronchoalveolar lavage (BAL) specimens taken from IPF patients are associated with earlier mortality.^20^ Neutrophil elastases (NE) are elevated in IPF BAL samples,^21^ and experimental data using murine models implicate NE in activation of the transforming growth factor-ß (TGF-ß) pathway and fibroblast proliferation.^22^ Indeed, lymphocytic aggregates are a recognised pathologic feature of IPF lesions.^18^ BAL specimens from IPF patients are also enriched for several T lymphocytes populations,^11^ and Th17 lymphocytes isolated from peripheral blood of patients with IPF have been identified as a source of TGF-β.^23^

The differences in statistical significance observed here between neutrophils, lymphocytes and monocytes in our multivariate analysis is probably a question of sensitivity. The HRs generated by our modelling should be interpreted as association between these immune cells and progression, which is statistically significant. Therefore, as a single point biomarker for progression of fibrosis, in established IPF (as opposed to very early disease), neutrophils and lymphocytes may be more sensitive than monocytes. Even more sensitive is NLR, which may well prove to be the biomarker for disease progression. NLR is a recognised surrogate marker of immune response, and a recognised correlate of disease severity, hospitalisation and mortality.^24 25^ A retrospective cross-sectional study demonstrated NLR, MLR and SIRI measurements to be greater in patients with IPF versus healthy controls,^26^ providing further support for our findings in progression of this disease.

Several limitations should be considered when interpreting the results of this analysis. The most obvious is the relatively small number of patients included, although compensated by more detailed characterisation and follow-up in the patients. The single-centre and retrospective nature of this study should also be taken forward by prospective, intervention and validation studies in a different cohort. Mortality and FVC decline reported in this study are higher than reported in other studies, most likely because of the long duration of follow-up. Also, study recruitment started before antifibrotic prescribing became available in the UK and, not unexpectedly in this cohort, baseline FVC% was lower in patients that died. However, strong points include statistical analyses including proportional hazards modelling of time-dependent covariates by multivariate analyses and longer median length of follow (31.0 months) will have adjusted for this. The effect of comorbidity burden, which is not insignificant in this and other IPF cohorts, on observed association between blood leucocytes and study outcomes is unknown. We also acknowledge that antifibrotic treatment and corticosteroid therapy may have affected blood leucocyte measurement, however, when adjusting for these covariates using multivariate regression significance was preserved (online supplemental table 1). In total only 11 patients within our cohort received Prednisolone (all prescribed for suspected acute exacerbation) during the 5-year follow-up. One patient was prescribed corticosteroids at the time of baseline assessment and prior blood draw. We do not believe this alters our interpretation of the data.

10.1136/bmjresp-2022-001202.supp1 Supplementary data

In summary, we report that neutrophil levels and lymphopaenia, and NLR measured from full blood count analysis were significantly associated with, and predictive of, FVC decline in this IPF cohort. Peripheral blood leucocyte measurements taken from full blood count analysis could be utilised to stratify IPF patients to those more likely to progress, and perhaps prioritised for anti-fibrotic treatment. This readily accessible measurement can also help inform prognosis, and identify patients for therapeutic trials.

## Data Availability

All data relevant to the study are included in the article or uploaded as online supplemental information.
