# Supplementary material for: Neutrophil lymphocyte ratio as an indicator for disease progression in Idiopathic Pulmonary Fibrosis
Source: BMJ Open Respir Res. 2022 Jun 16;9(1):e001202. doi: 10.1136/bmjresp-2022-001202 (PMC9207910; doi:10.1136/bmjresp-2022-001202)
Supplement: Supplementary data [file bmjresp-2022-001202supp001.pdf]

Supplementary file

Outcome [adjusted for age, gender, baseline FVC%, steroids]	HR (95% CI)	p value	p value PH assumption
Death			
Monocytes ($x10^9$/l)	1.57 (1.15-2.13)	0.004*	0.204
Lymphocytes ($x10^9$/l)	0.81 (0.52-1.26)	0.353	0.238
Neutrophils ($x10^9$/l)	1.21 (1.01-1.43)	0.038*	0.709
MLR	1.53 (1.02-1.95)	0.001*	0.123
NLR	1.24 (1.08-1.43)	0.002*	0.984
SIRI	1.08 (1.04-1.13)	0.001*	0.127
FVC decline>10%			
Monocytes ($x10^9$/l)	0.57 (0.09-3.54)	0.546	0.310
Lymphocytes ($x10^9$/l)	0.83 (0.52-1.34)	0.455	0.267
Neutrophils ($x10^9$/l)	1.32 (1.10-1.58)	0.003*	0.449
MLR	1.07 (0.69-1.64)	0.778	0.535
NLR	1.27 (1.13-1.42)	<0.001*	0.186
SIRI	1.03 (0.97-1.08)	0.338	0.149

Table S1. Multivariate Cox proportional hazard analysis for outcomes of survival, and lung function decline adjusted for age, gender, baseline FVC% and steroid use at clinic visit during follow up. For all multivariate models testing contribution of blood leukocytes against outcome these were all tested in combination [absolute monocyte, lymphocyte, and neutrophils] to explore interaction. For multivariate models exploring contribution of the leukocyte derived indexes [MLN, NLR or SIRI] these were tested individually and in absence of other leukocytes measurements or derived indexes. All adjusted covariates in each model satisfied the proportional hazard assumption. P values <0.05 are considered significant. MLR; monocyte/lymphocyte ratio, NLR; neutrophil/lymphocyte ratio, SIRI; systemic inflammatory response index.
